# Redetermination and absolute configuration of 7α-hy­droxy­royleanone

**DOI:** 10.1107/S1600536810020544

**Published:** 2010-06-05

**Authors:** Ibrahim Abdul Razak, Abdul Wahab Salae, Suchada Chantrapromma, Chatchanok Karalai, Hoong-Kun Fun

**Affiliations:** a X-ray Crystallography Unit, School of Physics, Universiti Sains Malaysia, 11800 USM, Penang, Malaysia; b Crystal Materials Research Unit, Department of Chemistry, Faculty of Science, Prince of Songkla University, Hat-Yai, Songkhla 90112, Thailand

## Abstract

The title compound [systematic name: 7α,12-dihy­droxy-8,12-abietadiene,11,14-dione or (4b*S*,8a*S*,10*R*)-3,10-dihy­droxy-2-isopropyl-4b,8,8-trimethyl-1,4,4b,5,6,7,8,8a,9,10-deca­hydro­phenanthrene-1,4-dione], C_20_H_28_O_4_, is an abietane diterpen­oid, which was isolated from the roots of *Premna obtusifolia *(Verbenaceae). Its crystal structure has been reported previously [Chen *et al.* (2000 ▶). *Jiegou Huaxue*, **19**, 122–125], but the absolute configuration could not be determined using data collected with Mo radiation. This redetermination using Cu radiation shows the the absolute configurations of the stereogenic centres at positions 4b, 8a and 10 to be *S*, *S* and *R*, respectively. Two intra­molecular O—H⋯O hydrogen bonds [one generating an *S*(5) ring and one generating an *S*(6) ring] and a number of short C—H⋯O contacts occur. In the crystal, mol­ecules are linked into infinite chains propagating in [100] by O—H⋯O hydrogen bonds and weak C—H⋯O inter­actions.

## Related literature

For background to Verbenaceae, diterpenes and their biological activity, see: Batista *et al.* (1994 ▶); Bunluepuech & Tewtrakul (2009 ▶); Jonathan *et al.* (1989 ▶); Kabouche *et al.* (2007 ▶); Kupchan *et al.* (1968 ▶, 1969 ▶); Nagy *et al.* (1999 ▶); Ulubelen *et al.* (2001 ▶). For the previous structure determination, see: Chen *et al.* (2000 ▶). For hydrogen-bond motifs, see: Bernstein *et al.* (1995 ▶) and for ring conformations, see: Cremer & Pople (1975 ▶). For bond-length data, see: Allen *et al.* (1987 ▶). For the stability of the temperature controller used in the data collection, see Cosier & Glazer (1986 ▶).
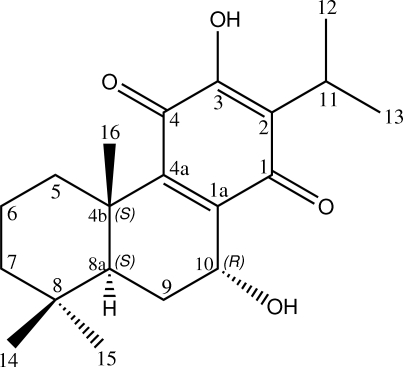


## Experimental

### 

#### Crystal data

C_20_H_28_O_4_
*M* *_r_* = 332.42
Orthorhombic, 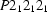

*a* = 7.6729 (1) Å
*b* = 9.3972 (1) Å
*c* = 24.1946 (3) Å
*V* = 1744.52 (4) Å^3^
*Z* = 4
Cu *K*α radiation
μ = 0.70 mm^−1^
*T* = 100 K
0.28 × 0.28 × 0.20 mm

#### Data collection

Bruker APEXII DUO CCD diffractometer
Absorption correction: multi-scan (*SADABS*; Bruker, 2009 ▶) *T*_min_ = 0.829, *T*_max_ = 0.871
6475 measured reflections
2578 independent reflections
2564 reflections with *I* > 2σ(*I*)
*R*_int_ = 0.015

#### Refinement

*R*[*F* ^2^ > 2σ(*F* ^2^)] = 0.027
*wR*(*F* ^2^) = 0.074
*S* = 1.04
2578 reflections
230 parameters
H atoms treated by a mixture of independent and constrained refinement
Δρ_max_ = 0.22 e Å^−3^
Δρ_min_ = −0.13 e Å^−3^
Absolute structure: Flack (1983 ▶), 970 Friedel pairs
Flack parameter: 0.13 (16)

### 

Data collection: *APEX2* (Bruker, 2009 ▶); cell refinement: *SAINT* (Bruker, 2009 ▶); data reduction: *SAINT*; program(s) used to solve structure: *SHELXTL* (Sheldrick, 2008 ▶); program(s) used to refine structure: *SHELXTL*; molecular graphics: *SHELXTL*; software used to prepare material for publication: *SHELXTL* and *PLATON* (Spek, 2009 ▶).

## Supplementary Material

Crystal structure: contains datablocks global, I. DOI: 10.1107/S1600536810020544/hb5468sup1.cif

Structure factors: contains datablocks I. DOI: 10.1107/S1600536810020544/hb5468Isup2.hkl

Additional supplementary materials:  crystallographic information; 3D view; checkCIF report

## Figures and Tables

**Table 1:** Hydrogen-bond geometry (Å, °)

*D*—H⋯*A*	*D*—H	H⋯*A*	*D*⋯*A*	*D*—H⋯*A*
O1—H1*O*1⋯O2^i^	0.88 (2)	2.24 (3)	2.9502 (15)	137 (2)
O1—H1*O*1⋯O4	0.88 (2)	2.52 (3)	2.9399 (14)	109.8 (19)
O3—H1*O*3⋯O2	0.83 (2)	2.075 (19)	2.5892 (14)	119.8 (19)
O3—H1*O*3⋯O4^ii^	0.83 (2)	2.42 (2)	3.1635 (14)	148.8 (18)
C1—H1*A*⋯O2	0.97	2.33	2.9493 (18)	121
C5—H5*A*⋯O1	0.98	2.52	2.9933 (17)	110
C7—H7*A*⋯O2^i^	0.98	2.42	3.0998 (17)	126
C15—H15*A*⋯O4	0.98	2.38	2.8549 (17)	109
C16—H16*C*⋯O3	0.96	2.58	3.1654 (19)	119
C17—H17*B*⋯O3	0.96	2.53	3.1204 (18)	119
C20—H20*A*⋯O2	0.96	2.51	3.1451 (18)	124

Symmetry codes: (i) 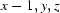; (ii) 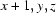.

**Fig. 1.**
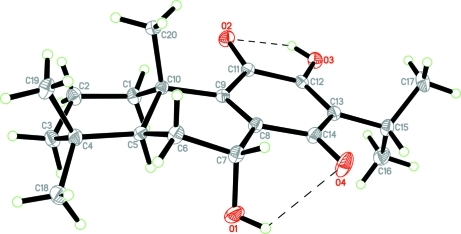
The structure of (I), showing 50% probability displacement ellipsoids. Intramolecular O—H···O hydrogen bonds are shown as dashed lines.

**Fig. 2.**
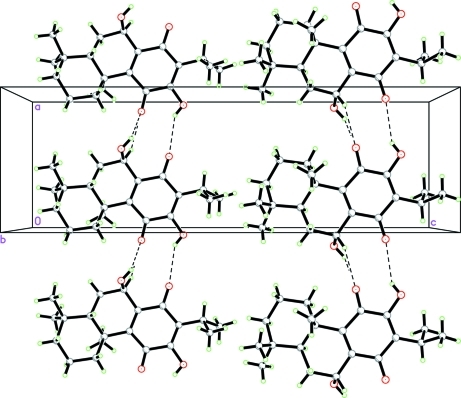
The crystal packing of (I) viewed along the b axis, showing one dimensional chains along the [1 0 0]. Hydrogen bonds are shown as dashed lines.

## supplementary crystallographic information

### Comment

The extracts of Verbenaceae plants were found to possess anti-HIV-1 integrase activity (Bunluepuech & Tewtrakul, 2009). *Premna obtusifolia* (Verbenaceae), a small tree found in the mangrove forests, is one of the Verbenaceae plants. As part of our study of chemical constituents and bioactive compounds from the roots of *Premna obtusifolia* (Verbenaceae) which were collected from Satun province in the southern part of Thailand, the title abietane diterpenoid (I) was isolated. It was known as horminone (Batista *et al.*, 1994) or 7α-hydroxyroyleanone (Nagy *et al.*, 1999) and the previous reports show that (I) exhibits significant biological activities as tumor inhibitors (Kupchan *et al.*, 1968, 1969; Jonathan *et al.*, 1989), antioxidant (Kabouche *et al.*, 2007) and antibacterial agents (Ulubelen *et al.*, 2001). The crystal structure of (I) has been reported (Chen *et al.*, 2000) but the absolute configuration could not be determined due to no large anomalous dispersion using a data set collected with Mo radiation. Our data of (I) was collected using Cu radiation with Bruker Apex-Duo CCD diffractometer and the absolute configuration at atoms C10, C5 and C7 (or positions 4b, 8a and 10 of abietane diterpenoid) were determined as *S*,*S*,*R* making use of the large anomalous scattering of Cu Kα*X*-radiation with the Flack parameter being refined to 0.13 (16). We report herein the crystal structure of (I) determined from the Cu data.

The molecule of (I) has three fused six membered rings (Fig. 1). The two cyclohexanes rings are *trans* fused. One cyclohexane ring (C1–C5/C10) is in a standard chair conformation whereas the other (C5–C10) is in half chair conformation, with the puckering parameter Q = 0.5419 (15) Å, θ = 51.68 (16)° and φ = 21.6 (2)° (Cremer & Pople, 1975). The benzoquinone ring (C8–C9/C11–C14/O2/O4) is slightly twisted with the maximum deviations of 0.060 (1) and -0.052 (1) Å for atoms C9 and C11, respectively. The O2, O3 and O4 atoms lie close to the mean plane of the C8–C9/C11–C14 ring with the *r.m.s.* of 0.0543 (1). The bond angles around C11 and C14 are indicative of *sp*^2^ hybridization for these atoms. The orientation of the propanyl group is described by the torsion angles C14–C13–C15–C17 =  -118.43 (14)° and C14–C13–C15–C16 = 116.53 (14)°. Intramolecular O1—H1O1···O4 and O3—H1O3···O2 hydrogen bonds (Table 1) generate S(6) and S(5) ring motifs, respectively (Fig. 1) (Bernstein *et al.*, 1995). The bond distances in (I) are within normal ranges (Allen *et al.*, 1987).

The crystal packing of (I) is stabilized by intermolecular O—H···O hydrogen bonds and weak C—H···O interactions (Fig. 2 and Table 1). The molecules are linked into infinite one dimensional chains along the [1 0 0] (Fig. 2) through O1—H1O1···O2 and O3—H1O3···O4 hydrogen bonds and weak C7—H7A···O2 interactions (Table 1).

### Experimental

The air-dried roots of *premna obtusifolia* (4.5 kg) were extracted with CH_2_Cl_2_ (2 × 20 *L*) at room temperature. The combined extracts were concentrated under reduced pressure to afford a dark yellow extract (40.5 g) which was subjected to quick column chromatography (QCC) over silica gel using solvents of increasing polarity from n-hexane to EtOAc to afford 12 fractions (F1—F12). Fraction F4 was further purified by QCC using hexane-acetone (9:1), yielding the title compound (57.5 mg). Yellow blocks of (I) were recrystallized from n-hexane after several days.

### Refinement

Hydroxy H atoms attached to O1 and O3 were located from the difference map and isotropically refined. The remaining H atoms were placed in calculated positions with (C—H) = 0.98 for CH, 0.97 for CH_2_ and 0.96 Å for CH_3_ atoms. The *U*_iso_ values were constrained to be 1.5*U*_eq_ of the carrier atom for methyl H atoms and 1.2*U*_eq_ for the remaining H atoms. A rotating group model was used for the methyl groups. The highest residual electron density peak is located at 0.78 Å from H7A and the deepest hole is located at 0.95 Å from C11. 970 Friedel pairs were used to determine the absolute configuration.

### Crystal data

**Table:**

C_20_H_28_O_4_	*F*(000) = 720
*M**_r_* = 332.42	*D*_x_ = 1.266 Mg m^−^^3^
Orthorhombic, *P*2_1_2_1_2_1_	Cu *K*α radiation, λ = 1.54178 Å
Hall symbol: P 2ac 2ab	Cell parameters from 2578 reflections
*a* = 7.6729 (1) Å	θ = 5.1–62.5°
*b* = 9.3972 (1) Å	µ = 0.70 mm^−^^1^
*c* = 24.1946 (3) Å	*T* = 100 K
*V* = 1744.52 (4) Å^3^	Block, yellow
*Z* = 4	0.28 × 0.28 × 0.20 mm

### Data collection

**Table:**

Bruker APEXII DUO CCD diffractometer	2578 independent reflections
Radiation source: sealed tube	2564 reflections with *I* > 2σ(*I*)
graphite	*R*_int_ = 0.015
φ and ω scans	θ_max_ = 62.5°, θ_min_ = 5.1°
Absorption correction: multi-scan (*SADABS*; Bruker, 2009)	*h* = −7→8
*T*_min_ = 0.829, *T*_max_ = 0.871	*k* = −10→10
6475 measured reflections	*l* = −27→27

### Refinement

**Table:**

Refinement on *F*^2^	Secondary atom site location: difference Fourier map
Least-squares matrix: full	Hydrogen site location: inferred from neighbouring sites
*R*[*F*^2^ > 2σ(*F*^2^)] = 0.027	H atoms treated by a mixture of independent and constrained refinement
*wR*(*F*^2^) = 0.074	*w* = 1/[σ^2^(*F*_o_^2^) + (0.0454*P*)^2^ + 0.3997*P*] where *P* = (*F*_o_^2^ + 2*F*_c_^2^)/3
*S* = 1.04	(Δ/σ)_max_ < 0.001
2578 reflections	Δρ_max_ = 0.22 e Å^−^^3^
230 parameters	Δρ_min_ = −0.12 e Å^−^^3^
0 restraints	Absolute structure: Flack (1983), 970 Friedel pairs
Primary atom site location: structure-invariant direct methods	Flack parameter: 0.13 (16)

### Special details

**Table:**

Experimental. The crystal was placed in the cold stream of an Oxford Cryosystems Cobra open-flow nitrogen cryostat (Cosier & Glazer, 1986) operating at 100.0 (1) K.
Geometry. All esds (except the esd in the dihedral angle between two l.s. planes) are estimated using the full covariance matrix. The cell esds are taken into account individually in the estimation of esds in distances, angles and torsion angles; correlations between esds in cell parameters are only used when they are defined by crystal symmetry. An approximate (isotropic) treatment of cell esds is used for estimating esds involving l.s. planes.
Refinement. Refinement of F^2^ against ALL reflections. The weighted R-factor wR and goodness of fit S are based on F^2^, conventional R-factors R are based on F, with F set to zero for negative F^2^. The threshold expression of F^2^ > 2sigma(F^2^) is used only for calculating R-factors(gt) etc. and is not relevant to the choice of reflections for refinement. R-factors based on F^2^ are statistically about twice as large as those based on F, and R- factors based on ALL data will be even larger.

### Fractional atomic coordinates and isotropic or equivalent isotropic displacement parameters (Å^2^)

**Table:**

	*x*	*y*	*z*	*U*_iso_*/*U*_eq_	
O1	0.39089 (14)	0.84016 (12)	0.26305 (4)	0.0253 (3)	
H1O1	0.310 (3)	0.830 (3)	0.2374 (11)	0.077 (9)*	
O2	1.09134 (13)	0.67838 (11)	0.21951 (4)	0.0216 (2)	
O3	1.04729 (13)	0.73182 (11)	0.11546 (4)	0.0209 (2)	
H1O3	1.132 (3)	0.720 (2)	0.1363 (8)	0.040 (6)*	
O4	0.44447 (13)	0.73046 (14)	0.15053 (4)	0.0300 (3)	
C1	0.98219 (19)	0.80709 (16)	0.32516 (6)	0.0198 (3)	
H1A	1.0903	0.7775	0.3079	0.024*	
H1B	0.9519	0.9001	0.3106	0.024*	
C2	1.00975 (19)	0.81849 (16)	0.38772 (6)	0.0228 (3)	
H2A	1.0508	0.7278	0.4018	0.027*	
H2B	1.0988	0.8892	0.3952	0.027*	
C3	0.8428 (2)	0.85964 (17)	0.41773 (6)	0.0233 (3)	
H3A	0.8124	0.9564	0.4076	0.028*	
H3B	0.8648	0.8584	0.4572	0.028*	
C4	0.68635 (19)	0.76239 (16)	0.40536 (6)	0.0204 (3)	
C5	0.66970 (18)	0.74674 (15)	0.34161 (5)	0.0173 (3)	
H5A	0.6466	0.8435	0.3284	0.021*	
C6	0.51347 (18)	0.66017 (16)	0.32182 (6)	0.0199 (3)	
H6A	0.5401	0.5594	0.3239	0.024*	
H6B	0.4137	0.6790	0.3453	0.024*	
C7	0.47085 (18)	0.70050 (16)	0.26259 (6)	0.0200 (3)	
H7A	0.3897	0.6310	0.2468	0.024*	
C8	0.63225 (18)	0.70964 (15)	0.22719 (6)	0.0181 (3)	
C9	0.79457 (18)	0.70681 (15)	0.24799 (5)	0.0158 (3)	
C10	0.83719 (18)	0.70011 (15)	0.31011 (6)	0.0169 (3)	
C11	0.93920 (17)	0.70252 (15)	0.20709 (6)	0.0171 (3)	
C12	0.90331 (18)	0.72713 (15)	0.14720 (6)	0.0168 (3)	
C13	0.74123 (19)	0.74250 (15)	0.12654 (6)	0.0182 (3)	
C14	0.59663 (18)	0.72713 (15)	0.16633 (6)	0.0188 (3)	
C15	0.70086 (18)	0.77406 (17)	0.06671 (6)	0.0211 (3)	
H15A	0.5738	0.7793	0.0632	0.025*	
C16	0.7741 (2)	0.91900 (17)	0.04969 (7)	0.0285 (4)	
H16A	0.7297	0.9912	0.0740	0.043*	
H16B	0.7397	0.9398	0.0124	0.043*	
H16C	0.8990	0.9169	0.0520	0.043*	
C17	0.7642 (2)	0.65721 (17)	0.02752 (6)	0.0260 (4)	
H17A	0.7154	0.5675	0.0386	0.039*	
H17B	0.8890	0.6519	0.0289	0.039*	
H17C	0.7278	0.6789	−0.0095	0.039*	
C18	0.5232 (2)	0.83806 (18)	0.42719 (6)	0.0266 (3)	
H18A	0.5417	0.8660	0.4649	0.040*	
H18B	0.4253	0.7746	0.4252	0.040*	
H18C	0.5004	0.9209	0.4051	0.040*	
C19	0.7033 (2)	0.62112 (17)	0.43667 (6)	0.0248 (3)	
H19A	0.6847	0.6372	0.4754	0.037*	
H19B	0.8178	0.5827	0.4310	0.037*	
H19C	0.6179	0.5551	0.4231	0.037*	
C20	0.8991 (2)	0.54667 (15)	0.32229 (6)	0.0202 (3)	
H20A	0.9797	0.5171	0.2942	0.030*	
H20B	0.8005	0.4838	0.3225	0.030*	
H20C	0.9554	0.5439	0.3577	0.030*	

### Atomic displacement parameters (Å^2^)

**Table:**

	*U*^11^	*U*^22^	*U*^33^	*U*^12^	*U*^13^	*U*^23^
O1	0.0192 (5)	0.0318 (6)	0.0248 (5)	0.0057 (5)	−0.0006 (5)	0.0037 (5)
O2	0.0130 (5)	0.0305 (6)	0.0214 (5)	0.0023 (4)	−0.0010 (4)	−0.0002 (5)
O3	0.0147 (5)	0.0290 (6)	0.0191 (5)	0.0004 (5)	0.0017 (4)	−0.0021 (5)
O4	0.0148 (5)	0.0531 (7)	0.0221 (5)	−0.0006 (5)	−0.0031 (4)	0.0068 (5)
C1	0.0162 (7)	0.0237 (7)	0.0194 (7)	−0.0028 (6)	0.0004 (6)	−0.0023 (6)
C2	0.0203 (7)	0.0257 (7)	0.0224 (7)	−0.0049 (7)	−0.0038 (6)	−0.0034 (6)
C3	0.0261 (8)	0.0248 (8)	0.0190 (7)	0.0001 (7)	−0.0014 (7)	−0.0037 (6)
C4	0.0194 (7)	0.0250 (8)	0.0168 (7)	0.0035 (6)	0.0000 (6)	0.0000 (6)
C5	0.0167 (7)	0.0185 (7)	0.0165 (6)	0.0012 (6)	−0.0003 (6)	0.0031 (6)
C6	0.0159 (7)	0.0256 (7)	0.0183 (7)	−0.0006 (6)	0.0021 (6)	0.0016 (6)
C7	0.0142 (6)	0.0268 (7)	0.0189 (7)	−0.0002 (7)	−0.0001 (6)	0.0019 (6)
C8	0.0168 (7)	0.0183 (7)	0.0192 (7)	−0.0001 (6)	−0.0008 (6)	0.0000 (6)
C9	0.0160 (7)	0.0140 (6)	0.0173 (7)	0.0003 (6)	0.0000 (6)	0.0010 (6)
C10	0.0138 (6)	0.0193 (7)	0.0177 (6)	0.0001 (6)	−0.0003 (6)	0.0003 (6)
C11	0.0160 (7)	0.0149 (7)	0.0205 (7)	−0.0008 (6)	−0.0005 (6)	−0.0026 (5)
C12	0.0163 (7)	0.0163 (6)	0.0178 (7)	−0.0003 (6)	0.0028 (6)	−0.0030 (6)
C13	0.0176 (7)	0.0184 (7)	0.0186 (7)	−0.0004 (6)	0.0007 (6)	−0.0023 (6)
C14	0.0159 (7)	0.0207 (7)	0.0198 (7)	0.0012 (6)	−0.0017 (6)	−0.0002 (6)
C15	0.0169 (7)	0.0291 (8)	0.0174 (7)	−0.0006 (7)	−0.0012 (6)	0.0005 (6)
C16	0.0341 (9)	0.0277 (8)	0.0237 (7)	0.0022 (8)	−0.0033 (7)	0.0057 (7)
C17	0.0295 (8)	0.0317 (8)	0.0169 (7)	−0.0059 (8)	0.0004 (7)	−0.0019 (6)
C18	0.0247 (8)	0.0348 (8)	0.0204 (7)	0.0048 (7)	0.0022 (6)	0.0003 (7)
C19	0.0257 (8)	0.0315 (8)	0.0171 (7)	−0.0025 (7)	−0.0007 (7)	0.0039 (6)
C20	0.0195 (7)	0.0211 (7)	0.0199 (7)	0.0019 (6)	−0.0028 (6)	−0.0007 (6)

### Geometric parameters (Å, °)

**Table:**

O1—C7	1.4488 (18)	C8—C9	1.344 (2)
O1—H1O1	0.88 (3)	C8—C14	1.5066 (19)
O2—C11	1.2266 (17)	C9—C11	1.4874 (19)
O3—C12	1.3461 (17)	C9—C10	1.5394 (18)
O3—H1O3	0.83 (2)	C10—C20	1.546 (2)
O4—C14	1.2288 (18)	C11—C12	1.4931 (19)
C1—C2	1.5322 (19)	C12—C13	1.348 (2)
C1—C10	1.543 (2)	C13—C14	1.476 (2)
C1—H1A	0.9700	C13—C15	1.5098 (19)
C1—H1B	0.9700	C15—C17	1.530 (2)
C2—C3	1.522 (2)	C15—C16	1.530 (2)
C2—H2A	0.9700	C15—H15A	0.9800
C2—H2B	0.9700	C16—H16A	0.9600
C3—C4	1.538 (2)	C16—H16B	0.9600
C3—H3A	0.9700	C16—H16C	0.9600
C3—H3B	0.9700	C17—H17A	0.9600
C4—C18	1.534 (2)	C17—H17B	0.9600
C4—C19	1.534 (2)	C17—H17C	0.9600
C4—C5	1.5547 (18)	C18—H18A	0.9600
C5—C6	1.526 (2)	C18—H18B	0.9600
C5—C10	1.5570 (19)	C18—H18C	0.9600
C5—H5A	0.9800	C19—H19A	0.9600
C6—C7	1.5178 (19)	C19—H19B	0.9600
C6—H6A	0.9700	C19—H19C	0.9600
C6—H6B	0.9700	C20—H20A	0.9600
C7—C8	1.5082 (19)	C20—H20B	0.9600
C7—H7A	0.9800	C20—H20C	0.9600
			
C7—O1—H1O1	101.4 (19)	C1—C10—C20	109.94 (11)
C12—O3—H1O3	107.0 (14)	C9—C10—C5	106.92 (11)
C2—C1—C10	112.22 (12)	C1—C10—C5	107.24 (11)
C2—C1—H1A	109.2	C20—C10—C5	115.00 (12)
C10—C1—H1A	109.2	O2—C11—C9	123.52 (13)
C2—C1—H1B	109.2	O2—C11—C12	116.22 (13)
C10—C1—H1B	109.2	C9—C11—C12	120.26 (12)
H1A—C1—H1B	107.9	O3—C12—C13	122.83 (12)
C3—C2—C1	111.88 (12)	O3—C12—C11	114.03 (12)
C3—C2—H2A	109.2	C13—C12—C11	123.13 (12)
C1—C2—H2A	109.2	C12—C13—C14	116.18 (12)
C3—C2—H2B	109.2	C12—C13—C15	124.46 (13)
C1—C2—H2B	109.2	C14—C13—C15	119.36 (13)
H2A—C2—H2B	107.9	O4—C14—C13	120.59 (13)
C2—C3—C4	114.40 (12)	O4—C14—C8	118.62 (13)
C2—C3—H3A	108.7	C13—C14—C8	120.78 (12)
C4—C3—H3A	108.7	C13—C15—C17	112.84 (12)
C2—C3—H3B	108.7	C13—C15—C16	110.95 (12)
C4—C3—H3B	108.7	C17—C15—C16	110.82 (12)
H3A—C3—H3B	107.6	C13—C15—H15A	107.3
C18—C4—C19	107.48 (12)	C17—C15—H15A	107.3
C18—C4—C3	107.13 (12)	C16—C15—H15A	107.3
C19—C4—C3	110.61 (12)	C15—C16—H16A	109.5
C18—C4—C5	108.58 (11)	C15—C16—H16B	109.5
C19—C4—C5	114.52 (12)	H16A—C16—H16B	109.5
C3—C4—C5	108.26 (11)	C15—C16—H16C	109.5
C6—C5—C4	115.24 (11)	H16A—C16—H16C	109.5
C6—C5—C10	110.17 (11)	H16B—C16—H16C	109.5
C4—C5—C10	116.38 (11)	C15—C17—H17A	109.5
C6—C5—H5A	104.5	C15—C17—H17B	109.5
C4—C5—H5A	104.5	H17A—C17—H17B	109.5
C10—C5—H5A	104.5	C15—C17—H17C	109.5
C7—C6—C5	109.42 (12)	H17A—C17—H17C	109.5
C7—C6—H6A	109.8	H17B—C17—H17C	109.5
C5—C6—H6A	109.8	C4—C18—H18A	109.5
C7—C6—H6B	109.8	C4—C18—H18B	109.5
C5—C6—H6B	109.8	H18A—C18—H18B	109.5
H6A—C6—H6B	108.2	C4—C18—H18C	109.5
O1—C7—C8	107.49 (11)	H18A—C18—H18C	109.5
O1—C7—C6	108.07 (12)	H18B—C18—H18C	109.5
C8—C7—C6	111.93 (12)	C4—C19—H19A	109.5
O1—C7—H7A	109.8	C4—C19—H19B	109.5
C8—C7—H7A	109.8	H19A—C19—H19B	109.5
C6—C7—H7A	109.8	C4—C19—H19C	109.5
C9—C8—C14	122.44 (12)	H19A—C19—H19C	109.5
C9—C8—C7	123.18 (12)	H19B—C19—H19C	109.5
C14—C8—C7	114.35 (12)	C10—C20—H20A	109.5
C8—C9—C11	116.29 (12)	C10—C20—H20B	109.5
C8—C9—C10	124.29 (12)	H20A—C20—H20B	109.5
C11—C9—C10	119.34 (12)	C10—C20—H20C	109.5
C9—C10—C1	110.91 (11)	H20A—C20—H20C	109.5
C9—C10—C20	106.82 (12)	H20B—C20—H20C	109.5
			
C10—C1—C2—C3	−56.85 (16)	C2—C1—C10—C5	55.46 (15)
C1—C2—C3—C4	54.11 (17)	C6—C5—C10—C9	52.09 (15)
C2—C3—C4—C18	−166.79 (12)	C4—C5—C10—C9	−174.34 (12)
C2—C3—C4—C19	76.36 (15)	C6—C5—C10—C1	171.09 (11)
C2—C3—C4—C5	−49.87 (16)	C4—C5—C10—C1	−55.33 (16)
C18—C4—C5—C6	−60.53 (17)	C6—C5—C10—C20	−66.31 (15)
C19—C4—C5—C6	59.57 (16)	C4—C5—C10—C20	67.26 (16)
C3—C4—C5—C6	−176.51 (12)	C8—C9—C11—O2	−168.73 (14)
C18—C4—C5—C10	168.22 (12)	C10—C9—C11—O2	8.2 (2)
C19—C4—C5—C10	−71.68 (16)	C8—C9—C11—C12	10.76 (19)
C3—C4—C5—C10	52.23 (16)	C10—C9—C11—C12	−172.31 (12)
C4—C5—C6—C7	157.64 (12)	O2—C11—C12—O3	−5.63 (19)
C10—C5—C6—C7	−68.21 (14)	C9—C11—C12—O3	174.85 (12)
C5—C6—C7—O1	−73.43 (14)	O2—C11—C12—C13	174.01 (13)
C5—C6—C7—C8	44.75 (16)	C9—C11—C12—C13	−5.5 (2)
O1—C7—C8—C9	107.73 (15)	O3—C12—C13—C14	176.88 (13)
C6—C7—C8—C9	−10.8 (2)	C11—C12—C13—C14	−2.7 (2)
O1—C7—C8—C14	−70.35 (15)	O3—C12—C13—C15	−3.3 (2)
C6—C7—C8—C14	171.12 (12)	C11—C12—C13—C15	177.11 (13)
C14—C8—C9—C11	−7.9 (2)	C12—C13—C14—O4	−175.35 (15)
C7—C8—C9—C11	174.19 (13)	C15—C13—C14—O4	4.8 (2)
C14—C8—C9—C10	175.36 (13)	C12—C13—C14—C8	5.73 (19)
C7—C8—C9—C10	−2.6 (2)	C15—C13—C14—C8	−174.11 (13)
C8—C9—C10—C1	−134.70 (14)	C9—C8—C14—O4	−179.11 (15)
C11—C9—C10—C1	48.64 (17)	C7—C8—C14—O4	−1.0 (2)
C8—C9—C10—C20	105.50 (16)	C9—C8—C14—C13	−0.2 (2)
C11—C9—C10—C20	−71.16 (15)	C7—C8—C14—C13	177.93 (13)
C8—C9—C10—C5	−18.10 (19)	C12—C13—C15—C17	61.75 (19)
C11—C9—C10—C5	165.24 (11)	C14—C13—C15—C17	−118.43 (14)
C2—C1—C10—C9	171.86 (12)	C12—C13—C15—C16	−63.29 (18)
C2—C1—C10—C20	−70.22 (15)	C14—C13—C15—C16	116.53 (14)

### Hydrogen-bond geometry (Å, °)

**Table:**

*D*—H···*A*	*D*—H	H···*A*	*D*···*A*	*D*—H···*A*
O1—H1O1···O2^i^	0.88 (2)	2.24 (3)	2.9502 (15)	137 (2)
O1—H1O1···O4	0.88 (2)	2.52 (3)	2.9399 (14)	109.8 (19)
O3—H1O3···O2	0.83 (2)	2.075 (19)	2.5892 (14)	119.8 (19)
O3—H1O3···O4^ii^	0.83 (2)	2.42 (2)	3.1635 (14)	148.8 (18)
C1—H1A···O2	0.97	2.33	2.9493 (18)	121
C5—H5A···O1	0.98	2.52	2.9933 (17)	110
C7—H7A···O2^i^	0.98	2.42	3.0998 (17)	126
C15—H15A···O4	0.98	2.38	2.8549 (17)	109
C16—H16C···O3	0.96	2.58	3.1654 (19)	119
C17—H17B···O3	0.96	2.53	3.1204 (18)	119
C20—H20A···O2	0.96	2.51	3.1451 (18)	124

Symmetry codes: (i) *x*−1, *y*, *z*;  (ii) *x*+1, *y*, *z*.
